# DNA Methyltransferase Inhibitors, Decitabine and Guadecitabine Overcome Immune-Checkpoint Blockade Resistance and Achieve Tumor Regression in the E0771 and 4T1 Murine Models of Triple-Negative Breast Cancer

**DOI:** 10.3390/cancers18152480

**Published:** 2026-08-02

**Authors:** S. Jennifer Wang, Carolyn Haynes, Laura Graham, Akhila Kunuthuru, Gina Tuzzolo, Anaya Surve, Madison Isbell, Jian He, Rebecca K. Martin, Harry Bear

**Affiliations:** 1 School of Medicine, Virginia Commonwealth University, Richmond, VA 23298, USA; wangsj4@vcu.edu (S.J.W.);; 2 Department of Surgery, Virginia Commonwealth University, Richmond, VA 23298, USA; 3 Division of Surgical Oncology, Department of Surgery and the Massey Comprehensive Cancer Center, Virginia Commonwealth University, Richmond, VA 23298, USA; 4 Flow Cytometry Shared Resource, Massey Comprehensive Cancer Center, Virginia Commonwealth University, Richmond, VA 23298, USA; isbellmg@vcu.edu (M.I.); rebecca.martin@vcuhealth.org (R.K.M.); 5 Biostatistics Shared Resource, Massey Comprehensive Cancer Center, Virginia Commonwealth University, Richmond, VA 23298, USA; hej7@vcu.edu; 6 Department of Microbiology and Immunology, Virginia Commonwealth University, Richmond, VA 23298, USA

**Keywords:** triple-negative breast cancer, immunotherapy, guadecitabine, decitabine, 4T1, E0771, DNA methyltransferase inhibitor

## Abstract

Background: Immune-checkpoint blockade (ICB) is a recent addition to the treatment options for breast cancer, especially the triple-negative (TNBC) subset. Inhibiting immunosuppressive factors in the tumor microenvironment (TME) and preventing or reversing T cell exhaustion may increase the likelihood of a response to ICB. We have shown that decitabine and guadecitabine, DNA methyltransferase inhibitors (DNMTi), prevent the systemic and TME accumulation of myeloid-derived suppressor cells (MDSCs), which are immunosuppressive. Results: Here, we show that adding DNMTi to the neoadjuvant + adjuvant ICB-based treatment of ICB-resistant 4T1 and E0771 murine tumors in Balb/C and C57Bl/6 mice, respectively, effectively reduced the tumor burden, with 52% of 4T1 tumors completely regressing across several studies. DNMTi-based therapy overcame ICB resistance (ICBR) in a selected subline of E0771, and treated tumors showed a reduction in MDSCs. We also show that, in E0771, DNMTi can overcome ICBR to multiple checkpoint inhibitors, and in 4T1, it can modulate anti-tumor immunity by enhancing central memory T cell (Tcm) formation and reducing T cell exhaustion. Conclusion: These pre-clinical findings support the further investigation of incorporating DNMTi as a new immunotherapy modality for TNBC.

## Simple Summary

Triple-negative breast cancer (TNBC) is an aggressive form of breast cancer with limited treatment options. Immune checkpoint blockade (ICB) can help the immune system fight cancer, but many TNBC tumors are resistant to ICB because the microenvironments are immunosuppressive. In this study, we tested whether adding DNA methyltransferase inhibitors (DNMTi) to ICB treatments can overcome this resistance in two mouse models of TNBC. We found that combining DNMTis with cyclophosphamide and ICB significantly shrank 4T1 tumors, with 52% disappearing completely. This combination was also effective in E0771 tumors which were selected for resistance to ICB alone. Tumors that were treated with DNMTi-containing regimens contained fewer immunosuppressive cells and more cell types that classically produce targeted immune responses and durable immune memory. Altogether, these findings suggest that DNMTi may alter TNBC, making them more responsive to existing ICB treatments.

## 1. Introduction

Breast cancer is the most common cancer and the second leading cause of cancer deaths for women in the United States [1]. Triple-negative breast cancers (TNBCs), which do not express significant levels of estrogen receptor (ER), progesterone receptor (PR), or human epidermal growth factor receptor 2 (HER2), have been associated with more aggressive disease and higher rates of mortality, especially in Black women, compared to other types of breast cancer [2,3].

Immune-checkpoint blockade (ICB) can induce strong and durable responses in many cancers, including breast cancer, but its current use in TNBC, combined with chemotherapy, is limited to early, high-risk disease or metastatic PD-L1-positive disease [4,5]. Furthermore, a significant proportion of patients do not respond to ICB alone or combined with chemotherapy, which likely reflects an immune-suppressed or “cold” tumor microenvironment (TME). ICB-responsive cancers tend to be “hot” tumors with the presence of functional anti-tumor CD8+ T cells or cytotoxic lymphocytes (CTLs), adequate antigen presentation, and accessible tumor antigen [6,7,8,9].

The TME of TNBC is often myeloid-dominant and immunosuppressive, characterized by the extensive infiltration of myeloid-derived suppressor cells (MDSCs) and tumor-associated macrophages (TAMs), especially type 2 TAMs. MDSCs enhance local tumor progression by suppressing CTLs directly and indirectly through promoting Treg differentiation, while also inducing T cell exhaustion [10,11]. Importantly, the accumulation of MDSCs in the TME has been associated with a poorer prognosis, later stage, and higher metastatic burden in patients with breast cancer [12,13]. Type 2 TAMs similarly shift CD4+ T cells towards Treg and Th2 phenotypes. Type 2 TAMs also directly impair CTLs and shift them away from the effector phenotype, and inhibit dendritic cell (DC) recruitment to the TME [14,15].

DCs are key orchestrators of anti-tumor immunity, as DC activation represents the major pathway of cross-presentation, priming naïve CD8+ T cells, and licensing CTL proliferation. Conversely, DC dysfunction or depletion is a hallmark of immune-cold tumors in which the absence of conventional DC (cDC)-mediated cross-presentation and T cell activation leads to reduced CD8+ T cell recruitment [16]. Consistent with this, many TNBC tumors are DC-poor or contain dysfunctional DCs, which inhibit T cell priming and responses to ICB [6,17]. Furthermore, tumor cells can also hypermethylate and thus downregulate the expression of antigenic and MHC genes to “camouflage” themselves from host immune surveillance and reduce antigen availability for presentation [18,19].

DNA methyltransferase inhibitors (DNMTi) such as guadecitabine (Gua) and decitabine (Dec), the active metabolite of Gua, may be capable of shifting the immunosuppressive TME of TNBC and restoring T cell functionality to improve responsiveness to ICB. Gua is resistant to degradation by cytidine deaminase, allowing for increased in vivo exposure of solid tumors to the active drug [20]. We previously found that treating tumor-bearing mice with Dec or Gua almost completely depleted MDSCs in the spleen in a 4T1 murine model of triple-negative breast cancer, which is poorly immunogenic and spontaneously metastasizing [19,21]. Time-course experiments by Luker et al. demonstrated that Gua prevents the accumulation of MDSCs, in part by depleting myeloid progenitors within the bone marrow [19]. In murine models, inhibiting or knocking out DNMT prevents T cell exhaustion and increases the in vivo persistence of therapeutically active T cells and responsiveness to ICB [22,23]. DNMTi has also been shown to upregulate MHC Class I expression in 4T1 cells, therefore having the potential to recover tumor antigen expression and presentation [19,21]. 

Here, we show that the representative DNMTi Gua and Dec increased the efficacy of ICB plus low-dose cyclophosphamide (Cyp) combination therapy when administered in the 4T1 murine model of TNBC. In some sense, CYP is a “holdover” from our prior studies of DNMTi augmentation of adoptive cellular therapy [19,21]. At the low doses used here, the primary effects of CYP, which is often used in clinical applications of cellular therapy, [24] are its mild lymphodepleting effect, which in turn induces secretion of cytokines that support lymphocyte proliferation, and its selective depletion of T regulatory cells [25,26]. When used prior to the surgical resection of primary tumors, the “triple-therapy” combination of DNMTi with ICB and Cyp prolonged metastasis-free and overall survival. DNMTi combined with dual ICB in the absence of Cyp also led to a reduction in the 4T1 tumor burden. Triple-therapy-treated tumors showed increased populations of CD8+ T cells, particularly central memory T cells (Tcm). We also report that Gua and Dec can each restore sensitivity to dual ICB in a resistant subline of E0771 tumors.

## 2. Materials and Methods

### 2.1. Cell Lines

No novel genetic sequence data were generated or deposited as part of this study. The cell lines used were commercially obtained. The 4T1 (ATCC CRL-259) and E0771 (CH3 Biosystems, Amherst, NY, USA, # 94A001) cell lines were purchased from American Type Culture Collection (ATCC) and CH3 Biosystems, respectively. 4T1 cells expressing luciferase (4T1-luc ATCC #CRL-2539-LUC2) were purchased from Gold Biotechnology. Immune-checkpoint blockade-resistant (ICBR) E0771 tumors were derived through serial passage in C57Bl/6J mice repeatedly treated with a combination of anti-PD1 and anti-CTLA4 until a stable ICBR phenotype was attained. Cells were cultured at 37 °C in DMEM media (Life Technologies, Carlsbad, CA, USA) supplemented with 10% (*v*/*v*) fetal bovine serum, 2 mmol/L L-glutamine (Gibco Thermo Fisher Scientific, Grand Island, NY, USA), 1% (*v*/*v*) penicillin/streptomycin (Gibco), 1% (*v*/*v*) MEM non-essential amino acids (Gibco), 1 mmol/L sodium pyruvate (Gibco), and 0.1 mmol/L 2-mercaptoethanol (Sigma-Aldrich, St. Louis, MO, USA).

### 2.2. Animal Strains

All animal protocols were approved by the Institutional Animal Care and Use Committee (IACUC) of Virginia Commonwealth University (VCU) (protocol #AM10256) and are in compliance with the NIH/NCI ethical guidelines for tumor-bearing animals. All mice were purchased from Envigo (Frederick, MD, USA): female Balb/cJ and C57Bl/6J. Mice aged 8–10 weeks and weighing an average of 20 g were selected for the experiments.

### 2.3. Subcutaneous Tumor Inoculations

A total of 50,000 4T1 or 100,000 E0771 cells were injected into animals’ left flanks or the 4th mammary fat pad (MFP) in sterile PBS. For experiments involving resection and follow-up for spontaneous metastases, 4T1-luc cells were suspended in Matrigel (Corning, Fisher Scientific, Bedford, MA, USA) or Cultrex (R&D Systems, Minneapolis, MN, USA)). Mice were randomized into different cages at the time of inoculation, with 5 mice per cage. The investigators were not blinded. The tumor size was measured 2–3 times weekly, and the tumor volume was calculated as [length/2 × width^2^]. Mice were euthanized by CO_2_ asphyxiation when tumors reached 2000 mm^3^ or had gross ulceration or when the mice showed weight loss or other signs of distress.

### 2.4. In Vivo Treatments Using DNMTi

All the appropriate groups received i.p. injections of guadecitabine (2.5 mg/kg) (gifted by Taiho Oncology, Inc., Princeton, NJ, USA) or decitabine (0.75 mg/kg) daily on days 7–10 post-inoculation, unless otherwise indicated. Those were found to be the highest tolerated doses without toxicity. Dec doses of 2mg/kg and 5mg/kg were tested in accordance with other studies [23] but were found to induce significant toxicity and fatality. A single dose of cyclophosphamide (VCU Pharmacy, Richmond, VA, USA) was injected i.p. at 100 mg/kg on day 7 unless stated otherwise. Anti-PD-1 (obtained from BioXCell, Lebanon, NH, USA) (100 mg) was injected i.p. every 3 days in 4 doses, starting on day 9, unless stated otherwise. For dual checkpoint blockade therapy, mice received anti-PD1 + anti-CTLA-4 or anti-LAG3 (all obtained from BioXCell), 100 micrograms i.p. every 3 days in 4 doses, starting on day 9, unless stated otherwise.

For resection/metastasis experiments, tumors were resected under inhalation general anesthesia (isoflurane) on day 13 after inoculation. The mice received 4 mg/kg meloxicam ER analgesic subcutaneously after the procedure and at 24 h postoperatively. The mice were assessed for recurrent tumor and distant metastases by imaging in an IVIS device every 3 days under inhalation anesthesia, within 10 min of the i.p. administration of 3mg/20g body weight Luciferin (Gold Biotechnology, Olivette, MO, USA)). The mice were euthanized when clinically appropriate. If complete resection of the primary tumor was not successful, those mice were excluded from subsequent evaluation of metastasis-free survival.

### 2.5. Flow Cytometry

Single-cell suspensions were obtained utilizing the gentleMACS tissue dissociator (Miltenyi Biotec, Gaithersburg, MD, USA) and stained with the fixable live/dead stain Zombie NIR (Biolegend, San Diego, CA, USA) as per the manufacturer’s instructions. Samples were then Fc-blocked with 2.4G2 for 5 min; surface antibodies (see below) were added and incubated for 30 min on ice. Samples stained with multiple fluorophores were stained in the presence of Brilliant Stain Buffer (BD Biosciences, Frederick, MD, USA) as per the manufacturer’s instructions. All cells were then fixed in 4% paraformalydehyde (PFA) fixation buffer (Biolegend). Flow data were collected using a Cytek Aurora (Cytek, Bethesda, MD, USA) with SpectraFlow acquisition software version 3.2.1 and analyzed with FlowJo (10.4.2). Total MDSCs were characterized as both monocytic and granulocytic populations combined. The gating for MDSC populations was as follows: CD11b+ Ly6Chi (monocytic-MDSCs) and CD11b+ Ly6CintLy6G+ (granulocytic MDSCs). Gating strategy is shown in Supplemental Figures S1 and S2 [27]. The antibodies used for flow cytometry were as follows: BUV395-CD45 (30-F11) and BUV496-F4/80 (T45-2342) from Fisher Scientific; eFluor450-MHCII (M5/114.15.2) and PerCP-eFluor710-CD206 (MR5F3) from eBiosciences (San Diego, CA, USA); and BV605-Ly6G (IA8), BV711-CD11c (N418), PE-Ly6c (HK1.4), and PE/Fire640-CD11b (M1/70) from Biolegend. APC-H2Kb (AF6-88.5) and PE-H2Db (KH95) were used for E0771, and PE-pan-MHCI was used for 4T1 experiments. All antibodies were used as per the manufacturers’ protocol.

### 2.6. Statistical Analysis

Each figure depicts 1 representative experiment of at least 3 independently conducted experiments. Group sizes were generally 5 animals per group; however, they could vary across experiments.

For each tumor-growth experiment, a single fixed assessment time point was selected as the latest day on which tumor-volume measurements were available for all mice before any mouse was euthanized for tumor size or other human endpoint. At the selected time point, mean tumor volume was compared across treatment and control groups using one-way ANOVA. When the omnibus ANOVA test indicated evidence of group differences, pairwise comparisons between each treatment group and the control were then performed using (Welch) two-sample *t*-tests. The resulting *p*-values were adjusted using the Holm’s (or Tukey’s) method to control the familywise Type I error rate.

Metastasis-free survival (MFS) was defined as the time from tumor implantation (day 0) until the development of metastatic disease or death. MFS was estimated using the Kaplan–Meier method and compared among groups using global log-rank tests. When pairwise log-rank tests were performed, the resulting *p*-values were adjusted using the Holm procedure. Mice without an observed event were administratively censored at their last assessment.

Flow cytometry endpoints were compared between control and triple-therapy groups at the selected days using unpaired two-sample *t*-tests.

All statistical analyses were performed using R version 4.1.x [28]. Unless stated otherwise, hypothesis tests were two-sided. Statistical significance was defined as *p* < 0.05 or adjusted *p* < 0.05 when correction for multiple comparisons was applied.

## 3. Results

### 3.1. Guadecitabine Increases the Efficacy of Single-Dose Cyclophosphamide and ICB in Reducing the Tumor Burden in Mice Bearing 4T1 Tumors

**Figure 1:**
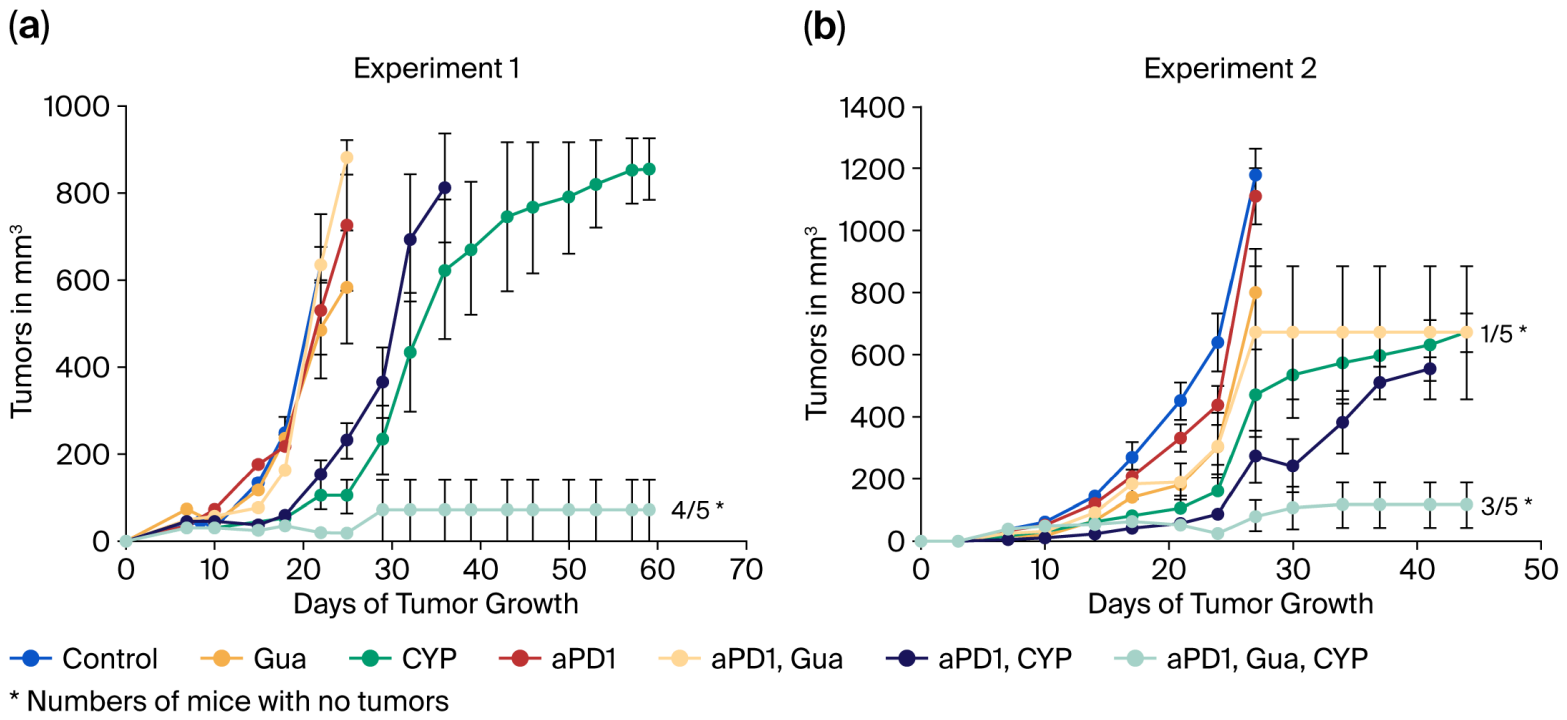
Balb/C mice were injected with 4T1 tumor cells on day 0 and then treated according to the regimens described in the text. Experiments 1 and 2 are shown in panels (**a**) and (**b**), respectively. Each experiment was analyzed separately at the latest assessment time points, i.e., day 22 for Experiment 1 and day 24 for Experiment 2. Mean tumor volume was compared across treatment and control groups using one-way ANOVA. Following the omnibus global test, pairwise comparisons between each treatment group and the control were then performed using Welch two-sample *t*-tests, with *p*-value adjusted for multiple testing. There were significant pairwise differences versus control for cyclophosphamide alone, anti-PD-1 + cyclophosphamide, and anti-PD-1 + guadecitabine + cyclophosphamide in both experiments. Also, we observed from the tumor-growth curves that these groups had lower tumor volumes than control at the analyzed time points. Fractions at the end of growth curves for some groups indicate the number of mice out of the total group whose tumors completely regressed.

Balb/C mice were injected in the 4th mammary fat pad with 50,000 4T1 cells on day 0 and then treated with 2.5 mg/kg Guad i.p. daily from days 7 to 10. Mice were treated with a single 100 mg/kg dose of cyclophosphamide on day 7 or 8, and 100 µg of anti-PD-1 was given on days 9, 12, 15, and 18. Two representative experiments are shown in Figure 1.

The latest assessment time points were day 22 for Experiment 1 and day 24 for Experiment 24. At these fixed time points, one-way ANOVA showed sufficient evidence of differences in mean tumor volume among treatment and control groups in both experiments (*p* < 0.0001). In Experiment 1, the pairwise comparisons versus control were statistically significant for cyclophosphamide alone (adjusted *p* = 0.0007), anti-PD-1 + cyclophosphamide (adjusted *p* = 0.0013), and anti-PD-1 + guadecitabine + cyclophosphamide (adjusted *p* = 0.0008). As shown in Figure 1a, these groups had lower tumor volumes than control on day 22. Additionally, mice treated with Guad, Cyp, and anti-PD-1 had tumors that never exceeded 45 mm^3^ in volume, and by day 22, four out of five tumors had completely regressed.

The tumor volumes in the triple-therapy group were lower than those in any other treatment group besides cyclophosphamide alone on day 32. While cyclophosphamide alone also achieved early tumor reduction, it did not exhibit durable control beyond day 30, with tumors in that group eventually reaching approximately the same volume as the control group at its peak. From day 32 onward, the only group with sustained tumor regression in this experiment was the triple-therapy group. By day 32, all the mice in groups besides the “triple-therapy” group, the anti-PD-1 + CYP group, and the CYP alone group had been euthanized, confirming much worse survival in the treatment arms without CYP or DNMTi. Therefore, both CYP and DNMTi confer a survival advantage but only DNMTi + ICB-containing therapy confers durable tumor control.

Experiment 2 shown in Figure 1b was performed under identical conditions to Experiment 1. Pairwise comparisons versus control on day 24 and the corresponding tumor growth curves were consistent with the findings from Experiment 1. Across five repeat experiments, the overall “cure rate” (mice with tumors measuring 0 mm^3^ for as long as they were observed) for mice treated with “triple-therapy” (guadecitabine, cyclophosphamide, and anti-PD-1) was 52%.

**Figure 2:**
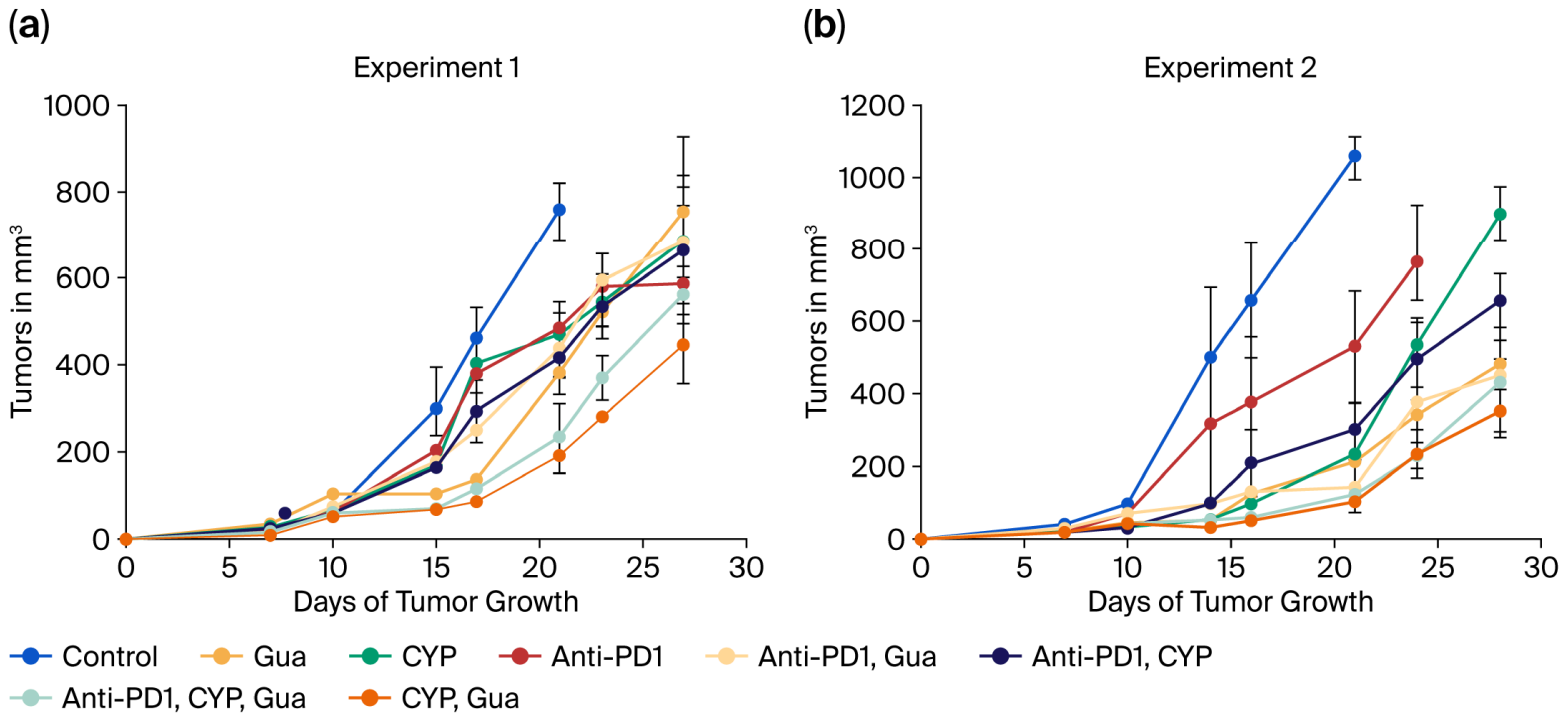
Athymic nude mice were injected with 4T1 tumor cells and treated according to the regimens outlined in the text. No mice in any treatment group achieved complete regression. No significant differences were found. (**a**,**b**) are duplicate experiments.

To understand the underlying cellular mechanism of triple therapy in tumor inhibition better, and to discern whether the efficacy of “triple therapy” relies on the presence of functional T cells, we repeated these tumor growth experiments in athymic nude mice. As shown in Figure 2, none of the treatments, whether alone or in combination, induced notable tumor regression in athymic nude mice. No mouse from any group achieved complete tumor regression. This lack of response shows not only that functional T cells are indispensable for the anti-tumor activity observed in immunocompetent 4T1-bearing mice treated with triple therapy, but also that the anti-tumor effects of DNMTi and Cyp in triple therapy are not exclusively a product of direct cytotoxicity or depletion of the myeloid compartment alone.

### 3.2. DNMTi + ICB and CYP Improves Metastasis-Free Survival in Neoadjuvant 4T1 Studies

We next sought to investigate the effect of Gua on metastasis-free survival when given in the neoadjuvant setting, starting prior to the resection of the primary tumors. Balb/C mice were injected in the flank with 100,000 4T1-luc cells suspended in either Cultrex or Matrigel and treated with 2.5 mg/kg Gua on days 7–10; 100 mg/kg cyclophosphamide on day 7 or 8; and 100 mg of anti-PD-1 on days 9, 12, 15, and 18. Tumor resection occurred on day 13.

**Figure 3:**
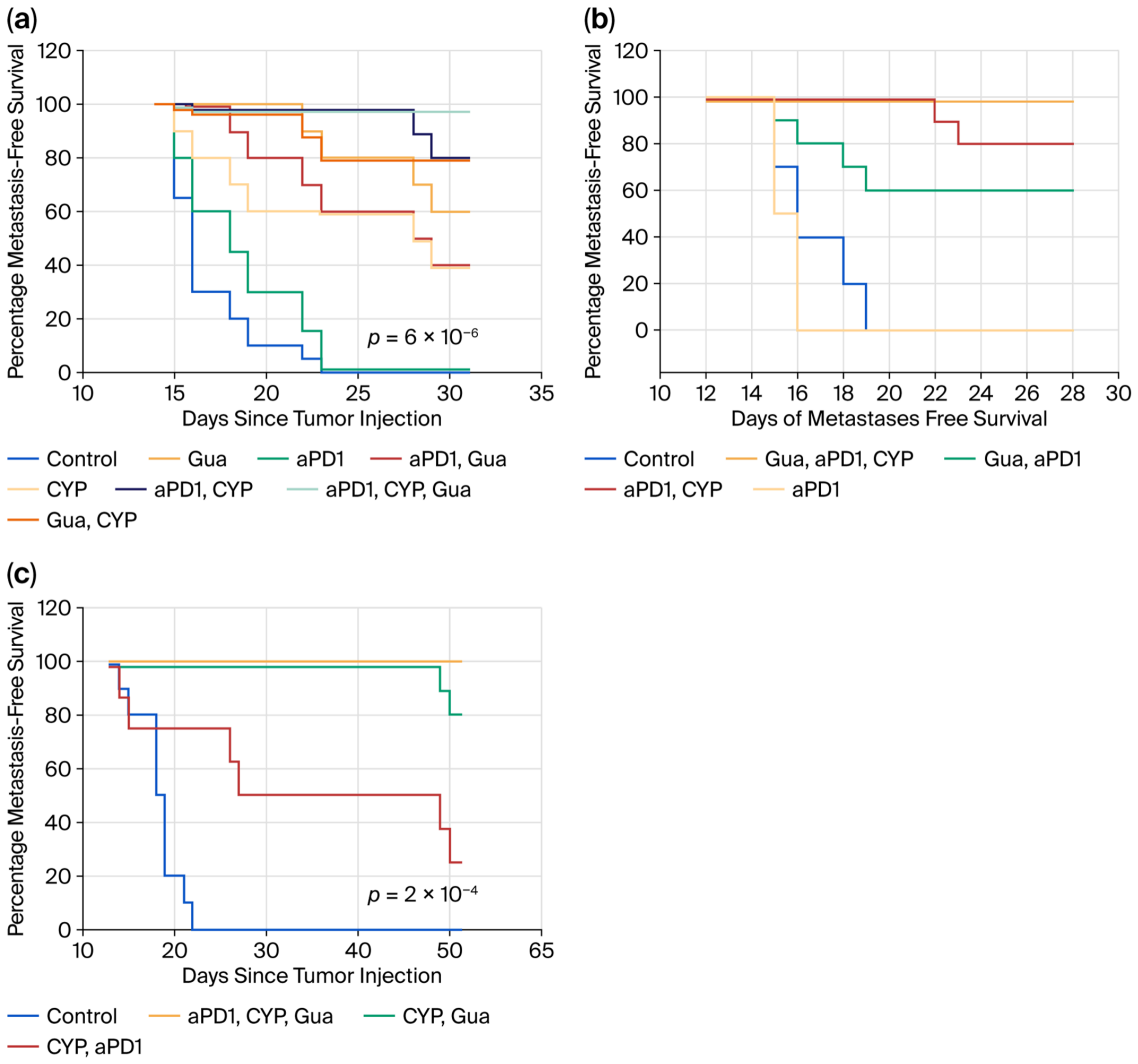
Metastasis-free survival experiment in Balb/C mice injected with 4T1-luciferase tumor cells. (**a**) All treatment regimens; 2.5 mg/kg Gua was administered on days 7–10; Cyp (100 mg/kg) on day 7; and anti-PD-1 (100 µg) on days 9, 12, 15, and 18. (**a**) 4T1-luciferase cells were injected in the flank with significantly longer MFS in triple-therapy-treated mice (*p* < 0.000006). (**b**) Repeat experiment similar to (**a**). Metastasis-free survival differed significantly among treatment groups according to the global log-rank test (*p* = 0.0001). Pairwise log-rank comparisons identified a difference in MFS between triple-therapy-treated mice and control (adjusted *p* = 0.023); the observed Kaplan–Meier curve for triple therapy was above that for control. (**c**) 4T1-luciferase tumor cells, injected into the mammary fat pad directly utilizing an open-injection technique. 4T1-luciferase cells were injected in the flank with significantly longer MFS in triple-therapy-treated mice (*p* < 0.0002).

Kaplan–Meier curves of metastasis-free survival from two flank-injection experiments are shown in Figure 3a,b. In both experiments, treatment with anti-PD-1 alone in the 4T1 model showed little apparent improvement in metastasis-free survival compared to controls (Figure 3a,b). Adding DNMTi and/or CYP to ICB substantially augmented metastasis-free survival. Furthermore, we observed 100% metastasis-free survival by day 32 in mice receiving “triple therapy”. This was repeated across four experiments, with an overall metastasis-free survival rate of 90% among mice receiving “triple therapy”.

**Figure 4:**
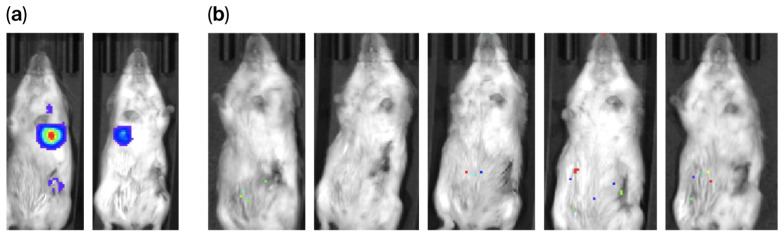
Representative IVIS imaging of mice 23 days after injection with 4T1-luciferase cells. (**a**) Control mice with visible lung metastases. The other three mice from the control group were euthanized because of lung metastases which resulted in mice reaching humane endpoints 16 days after injection with 4T1-luciferase cells. (**b**) Mice treated with anti-PD-1, cyclophosphamide, and Gua, with 100% metastasis-free survival on day 23.

When performing these metastasis-free survival experiments, we injected tumor cells into the flank because we found that injecting tumor cells into the mammary fat pad (MFP) resulted in more incomplete resections of the primary tumors, thus obscuring the data from metastasis-free survival studies. To ensure the reproducibility of the 4T1-luciferase cell injection into the MFP, we repeated the treatment protocol with mice that were given open injections of 4T1-luciferase cells under direct vision into the MFP. With this approach, we found results comparable to those of our studies utilizing flank injections (Figure 3c). Figure 4 shows representative IVIS scans of control mice and mice treated with triple therapy.

### 3.3. Effects of “Triple Therapy” with DNMTi + CYP + ICB on 4T1 Immune Tumor Microenvironment

**Figure 5:**
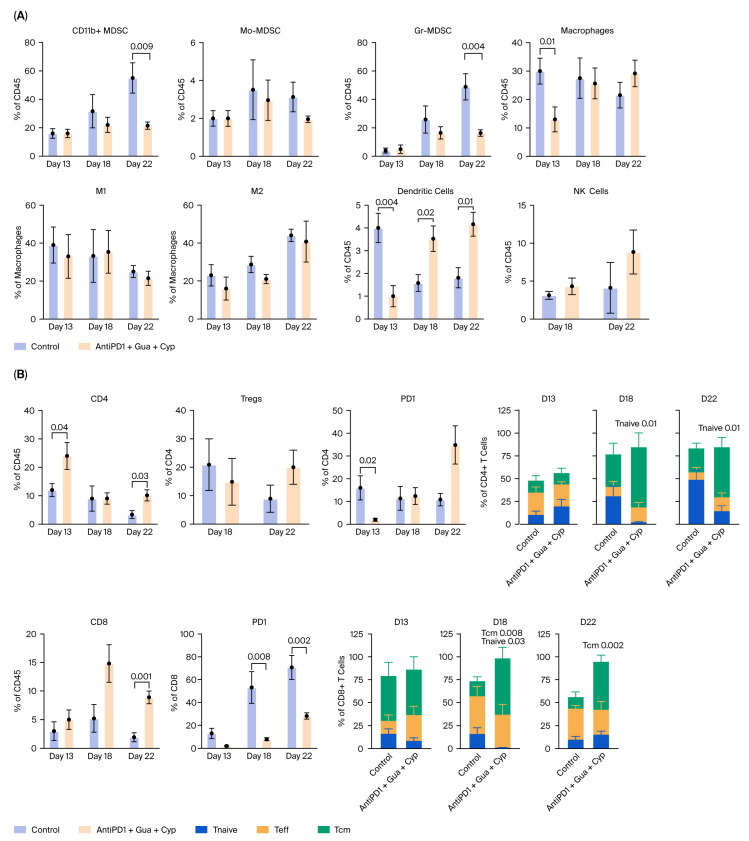
Results from flow cytometry performed on cells from 4T1 tumors excised from Balb/C mice comparing control mice to mice treated with “triple therapy”—guadecitabine, anti-PD-1, and a single low dose of cyclophosphamide. Unpaired two-sample *t*-tests were used to assess significance. (**A**) Comparing macrophages, monocytic MDSCs, and granulocytic MDSCs as a percentage of CD45+ cells. (**B**) Comparing CD4+ and CD8+ activation and differentiation states.

To characterize the impact of triple therapy on the TME of 4T1 tumors over time, flow cytometry was performed on tumors excised on days 13, 18, and 22. Though DNMTi are known to reduce systemic MDSC accumulation, the temporal effect of triple therapy on intratumoral MDSCs was difficult to anticipate. Flow cytometry performed on tumors from mice treated with triple therapy of Gua, anti-PD1, and Cyp in fact showed a significant decrease in MDSCs as a fraction of total CD45+ (*p* = 0.0057), especially granulocytic MDSCs (Gr-MDSCs) on day 22 (*p* = 0.0049) (Figure 5A). When administered individually, these three agents have previously been found to have different effects on macrophage polarization; our experiments with triple therapy showed a statistically significant decrease in macrophages, affecting both M1 (*p* = 0.035) and M2 subsets (*p* = 0.011), but only on day 13.

Total T cells, CD4+ T cells, and CD8+ T cells as percentages of all CD45+ cells did not increase substantially by D18. However, an increase in all three was present by day 22 and was significant in the triple-therapy group compared to controls (total T cells: *p* = 0.00017; CD4+ of CD45: *p* = 0.0286; CD8+ of CD45: *p* = 0.00090) (Figure 5B). Subset analysis on days 18 and 22 further revealed significant increases in the frequencies of naïve CD4+ (*p*= 0.01) and naïve (*p* = 0.03) CD8+ cells in triple-therapy-treated mice compared to controls. Central memory (Tcm) CD8+ cells were also significantly higher in the triple-therapy-treated mice than control mice on both day 18 (*p* = 0.008) and day 22 (*p* = 0.002). PD1+/CD8+ T cells were significantly lower in the triple-therapy mice compared to controls on both day 18 (*p* = 0.031) and day 22 (*p* = 0.021). Within the treatment group, PD-1^+^ CD8^+^ T cells increased significantly from day 18 to day 22 (*p* = 0.0002), whereas no significant change was observed in controls. This is consistent with the known DNMTi effect of the epigenetic reversal of T cell exhaustion. No significant difference was found in the proportion of effector T cells (Teff) of either T cell subtype at any time point. This suggests that the shift in adaptive immunity was towards more durable memory, or Tcm, rather than terminal effector differentiation. This overall timeline of T cell activation following a delayed suppression of the myeloid compartment is consistent with previous descriptions of DNMTi effects [21,29,30]. We also observed a significant and consistent rise in intratumoral CD11+ DCs on days 18 (*p* = 0.023) and 22 (*p* = 0.014) compared to controls.

### 3.4. Guadecitabine and Decitabine Were Associated with Restored Responsiveness to Immune-Checkpoint Blockade in Mice Bearing ICB-Resistant E0771 Tumors

**Figure 6:**
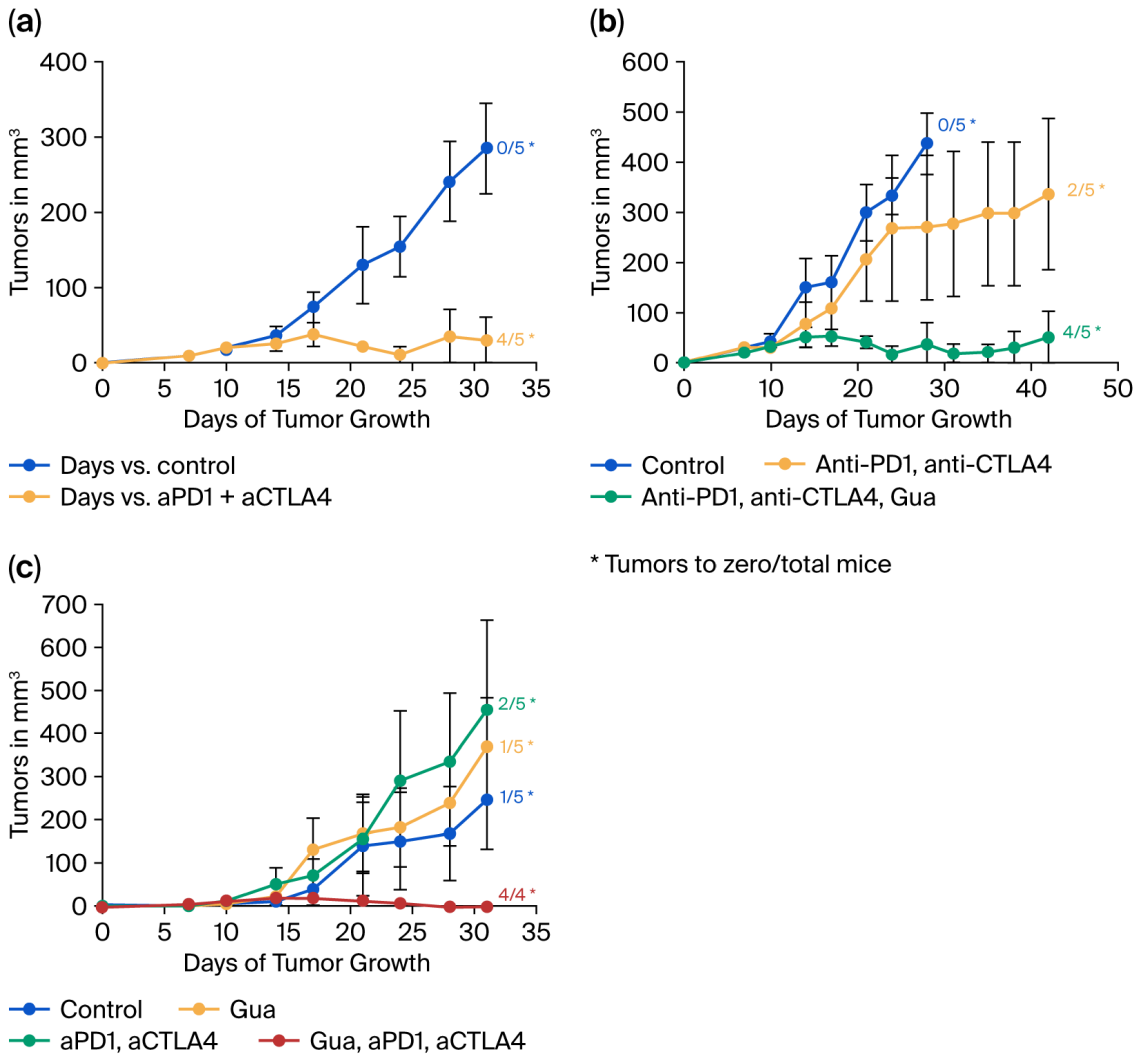
Tumor growth experiments utilizing parental and ICBR E0771 cell lines. Fractions at the ends of growth curves for some groups indicate the number of mice out of the total group whose tumors completely regressed. (**a**) Effect of treating parental line E0771 with dual immune-checkpoint blockade with anti-PD-1 + anti-CTLA4, 100 mg i.p. of each given every 3 days four times, starting on day 9. (**b**) Treatment of ICB-resistant (ICBR) E0771 with the same regimen of dual ICB +/- Gua (2.5 mg/kg i.p. daily four times, starting on day 7). Mice treated with triple therapy showed significant decreases in tumor volume (*p* < 0. 0001). (**c**) Same treatment as (**b**) with significant difference in triple-therapy-treated tumors (*p* = 0. 0008).

To study how DNMTi could alter the TME and increase the efficacy of ICB in another TNBC model, we sought to create an ICB-resistant subline of E0771. Several groups have characterized the TME to identify TME subtypes which prove to be more or less immunosuppressive. Kim et al. described a neutrophil-enriched subtype of tumors that tend to be resistant to ICB, as opposed to their macrophage-enriched counterparts, which had a more variable response to ICB [31]. As described by others, when mice bearing E0771 tumors were treated with dual ICB (anti-PD-1 plus CTLA4), the tumors in most mice grew significantly more slowly than those in controls and most regressed completely. We have also replicated this with the wildtype E0771 (Figure 6a). By selecting the rare “outlier” tumors that progressed despite dual ICB and serially passaging and re-selecting resistant tumors in subsequent generations, as described previously by others [31,32], we were able to develop a stably ICB-resistant (ICBR) subline of E0771 which did not respond to anti-PD1 and anti-CTLA4. When Gua (2.5 mg/kg i.p. on days 7–10) was added to dual ICB, these otherwise ICBR tumors grew significantly more slowly than either control tumors or tumors treated with dual ICB alone, with an overall “cure rate” of 64% across seven experiments and a significant reduction in tumor volumes compared to controls (*p* = 0.03). These results, illustrated in Figure 6b,c, show that DNMTi can overcome tumor resistance to immune-checkpoint blockade. Notably, Gua alone had no significant effect on E0771 tumor growth.

**Figure 7:**
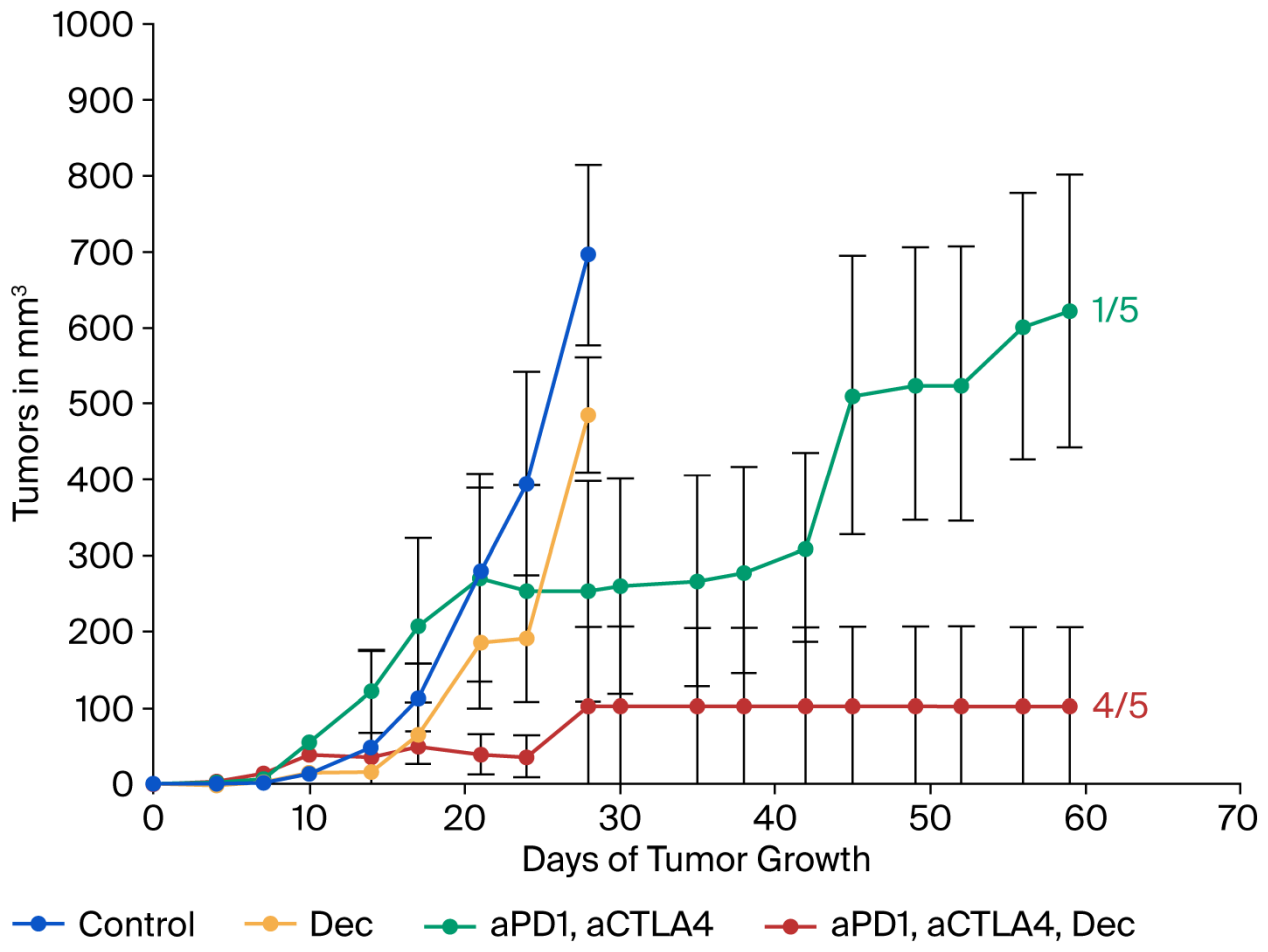
C57BL/6 mice were injected with ICBR E0771 tumor cells on day 0. Decitabine (0.75 mg/kg i.p.) was given on days 7–10; anti-PD-1 and anti-CTLA4 were given on days 9, 12, 15, and 18. Fractions shown at the ends of growth curves for each group are the numbers of mice with complete tumor regression out of the total n in each group. Treatment with Dec + aPD1 + aCTLA4 induced complete tumor regression (*p* = 0.0008).

**Figure 8:**
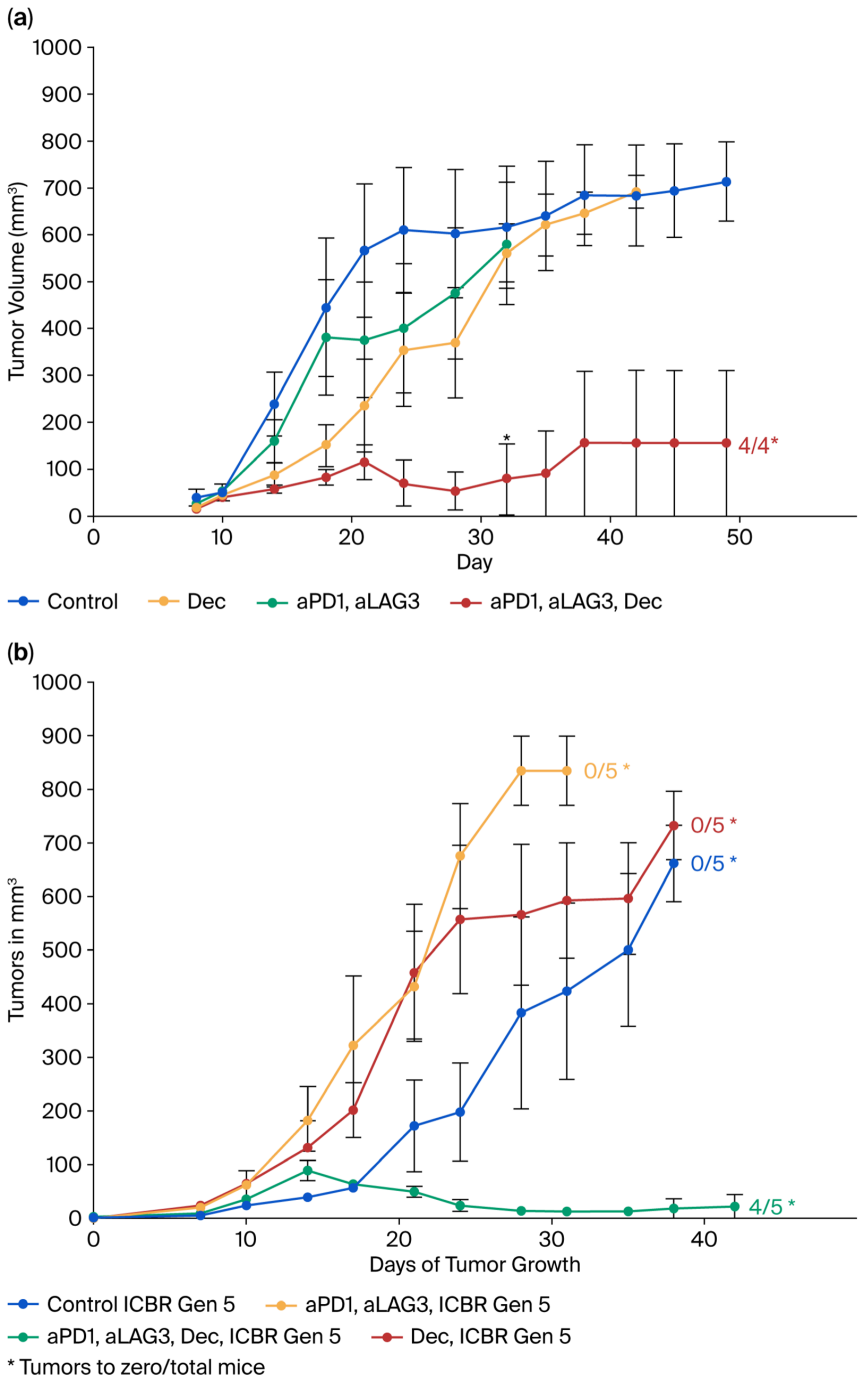
E0771 tumor growth curves in C57Bl/6 mice. (**a**) Mice were injected with ICBR E0771 tumor cells on day 0. Decitabine was given at 1mg/kg on days 7–9 and 14–16; anti-PD-1 and anti-LAG-3 (100ug) were given on days 9, 12, 15, and 18. Although the observed differences between the Dec + anti-PD-1 + anti-LAG3 group and each of the other three groups at the selected days was numerically substantial, the unpaired two-sample *t*-test did not provide sufficient evidence of group differences. Given the small group sizes, the estimated differences were imprecise, and the result should be interpreted cautiously. (**b**) Mice were injected with ICBR E0771 ICBR on day 0. Antibody treatments were the same as in (a), except that Dec was given at 0.75 mg/kg i.p. on days 7–10; fractions shown for each group represent the number of mice with complete tumor regression out of the total n in each group. Treatment with Dec + aLAG3 + aPD1 induced significant regression of ICBR E0771 (*p* < 0.0001).

We also repeated these experiments utilizing Dec rather than Gua. Similar to our results with Gua, we found that combination treatment with Dec and dual ICB induces complete regression in 70% of mice bearing ICBR E0771 tumors (*p* = 0.0008) (Figure 7). We found that this ICBR E0771 subline was resistant to treatment with anti-PD-1 + anti-LAG-3 (Figure 8). Interestingly, adding Dec to this combination ICB overcame resistance, with 4/5 mice achieving complete tumor regression.

Further studies with the ICBR E0771 also showed that anti-LAG3-containing dual ICB combined with anti-PD-1 and Dec induced significant and persistent reductions in tumor growth (Figure 8a,b).

**Figure 9:**
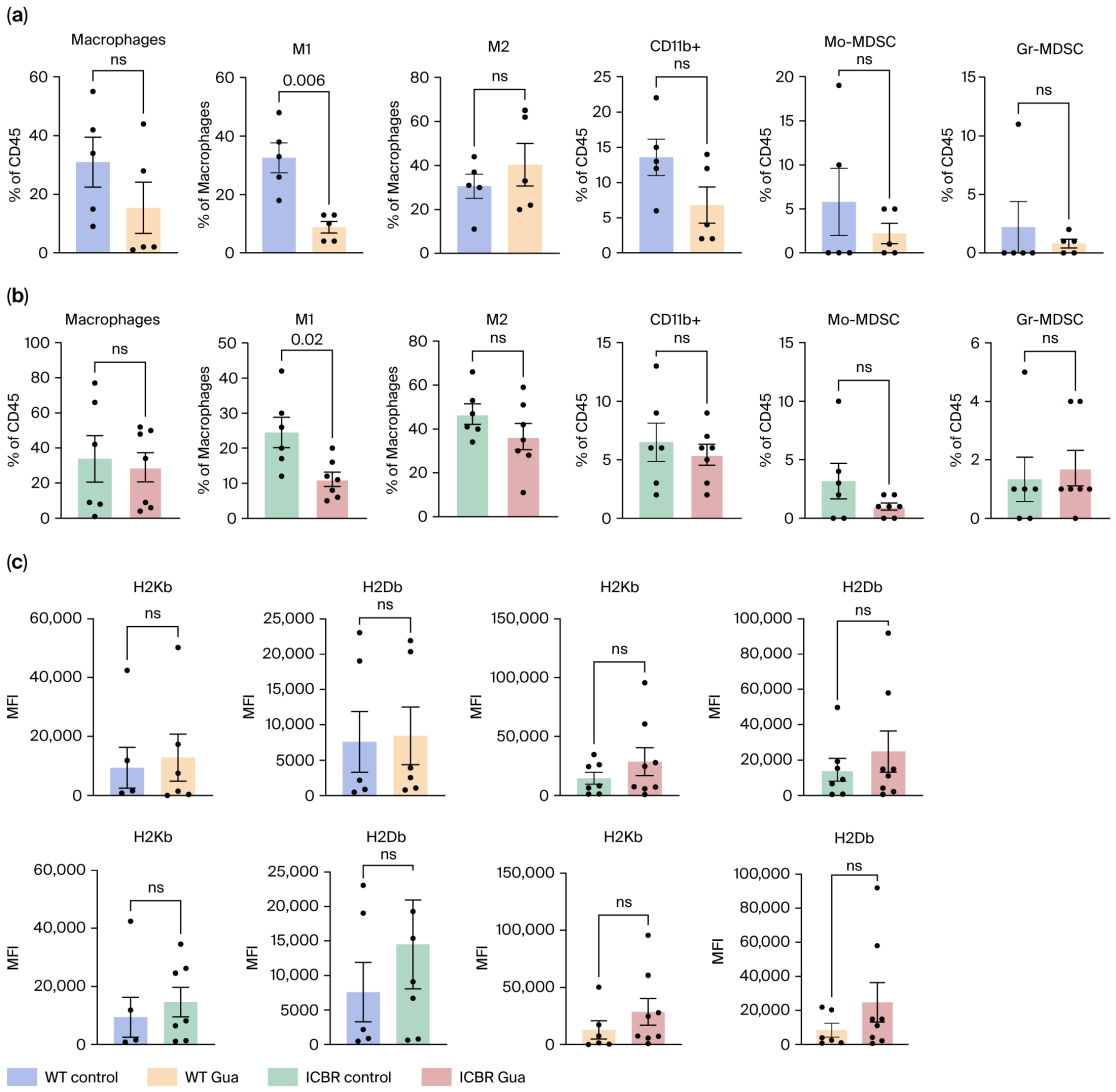
Flow cytometry results from E0771 tumors; *t*-tests were used to calculate significance. (**a**) Count of macrophages in control vs. treated mice, compared between ICBR and wildtype E0771 tumors. (**b**) Count of MDSCs. (**c**) MFI of H-2Kb (MHC Class I). In (**b**,**c**), although observed between-group differences were numerically substantial for these comparisons, the unpaired two-sample *t*-test did not provide sufficient evidence of group differences. Given the small group sizes, the estimated differences were imprecise, and the non-significant (ns) results should be interpreted cautiously.

To characterize the effects of DNMTi-containing ICB regimens on the E0771 TME, we then performed flow cytometry to investigate the potential mechanisms by which Gua overcomes resistance to immune-checkpoint blockade in E0771 tumors. We found that, at baseline, ICBR tumors had more M2s and monocytic MDSCs and fewer DCs than wildtype (WT) tumors, suggesting that the acquired resistance may be attributable to an immunosuppressive TME (Figure 9). The count of M1 macrophages was also significantly decreased in Guad-treated mice compared to control, regardless of whether mice were bearing WT or ICBR E0771 tumors. We also observed a statistically non-significant increase in Mean Fluorescence Intensity (MFI) of H-2Kb (MHC Class I) in tumor cells (negative for CD45) in wildtype and ICBR tumors from mice treated with Gua. In both the wildtype and ICBR E0771 models, we found that neither Gua nor Dec substantially decreased macrophage or mo-MDSC populations on day 13 but rather consistently increased DC populations. Therefore, DNMTi may restore responsiveness to ICB by enhancing antigen-presenting capacity by means of DC expansion to increase functional T cell activity.

**Figure 10:**
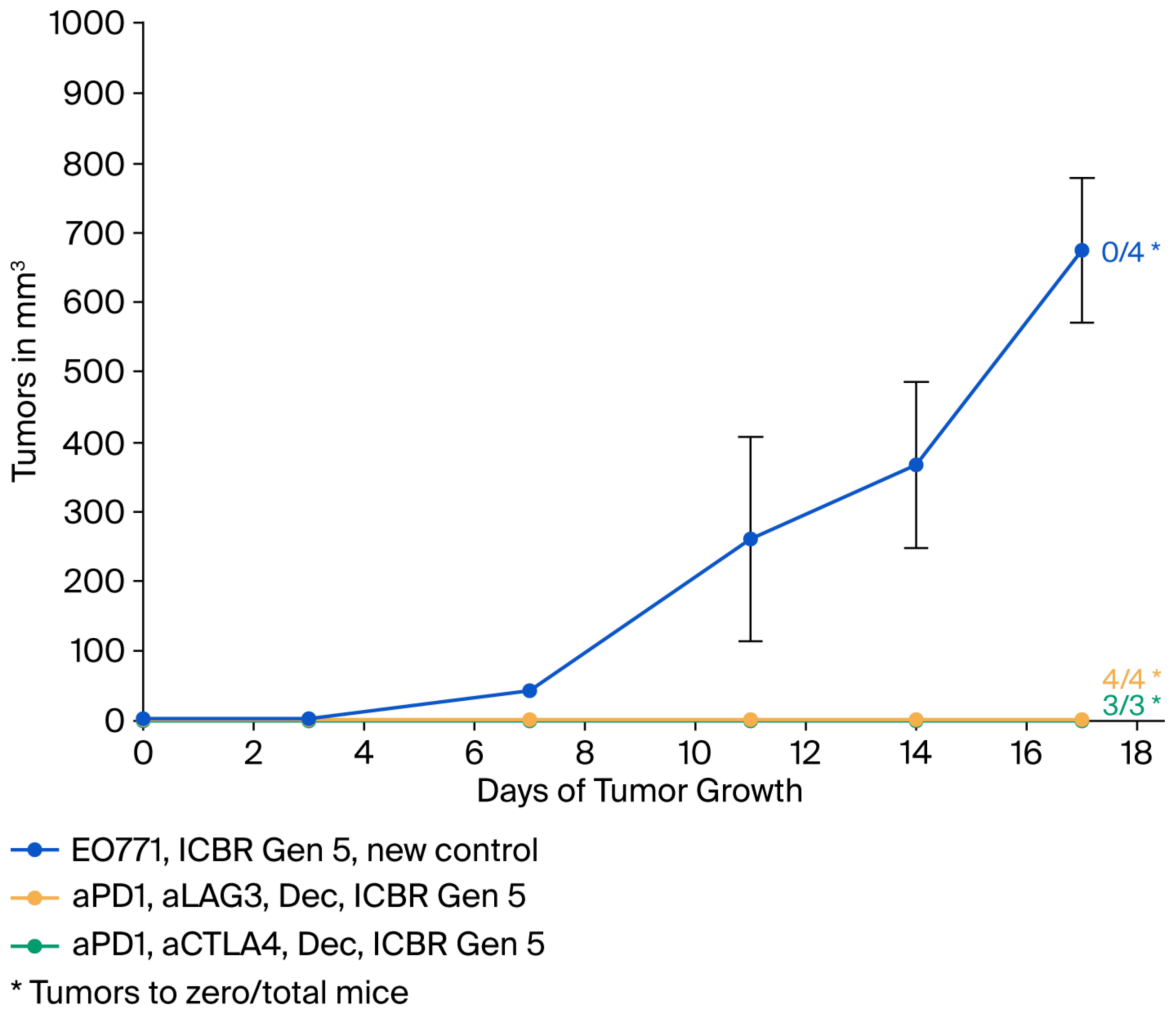
Mice that were initially cured from ICBR E0771 tumors by either combination of ICB antibodies + Dec were rechallenged on the opposite-side MFP. Fractions shown in the legend for each group are the numbers of mice with complete tumor regression out of the total n in each group. None of these mice received any treatment; 100% of mice treated with dual ICB + Dec resisted rechallenge (*p* < 0.0001).

We also wanted to establish whether mice cured from ICBR E0771 tumors with dual-ICB and DNMTi (Dec, in this case) would be resistant to tumor rechallenge. We injected 200,000 E0771 tumor cells into the opposite-side MFP of mice that were originally cured with DNMTi + dual ICB and a group of four naïve control mice. In both groups previously cured with dual-ICB + Dec, 100% of the mice were resistant to tumor rechallenge (*p* < 0.0001), compared to 0% of the control mice (Figure 10). Notably, no treatment was provided during the tumor rechallenge.

### 3.5. Inhibition of 4T1 Tumor Growth Using Chemotherapy-Free Regimens of Dual ICB + DNMTi

**Figure 11:**
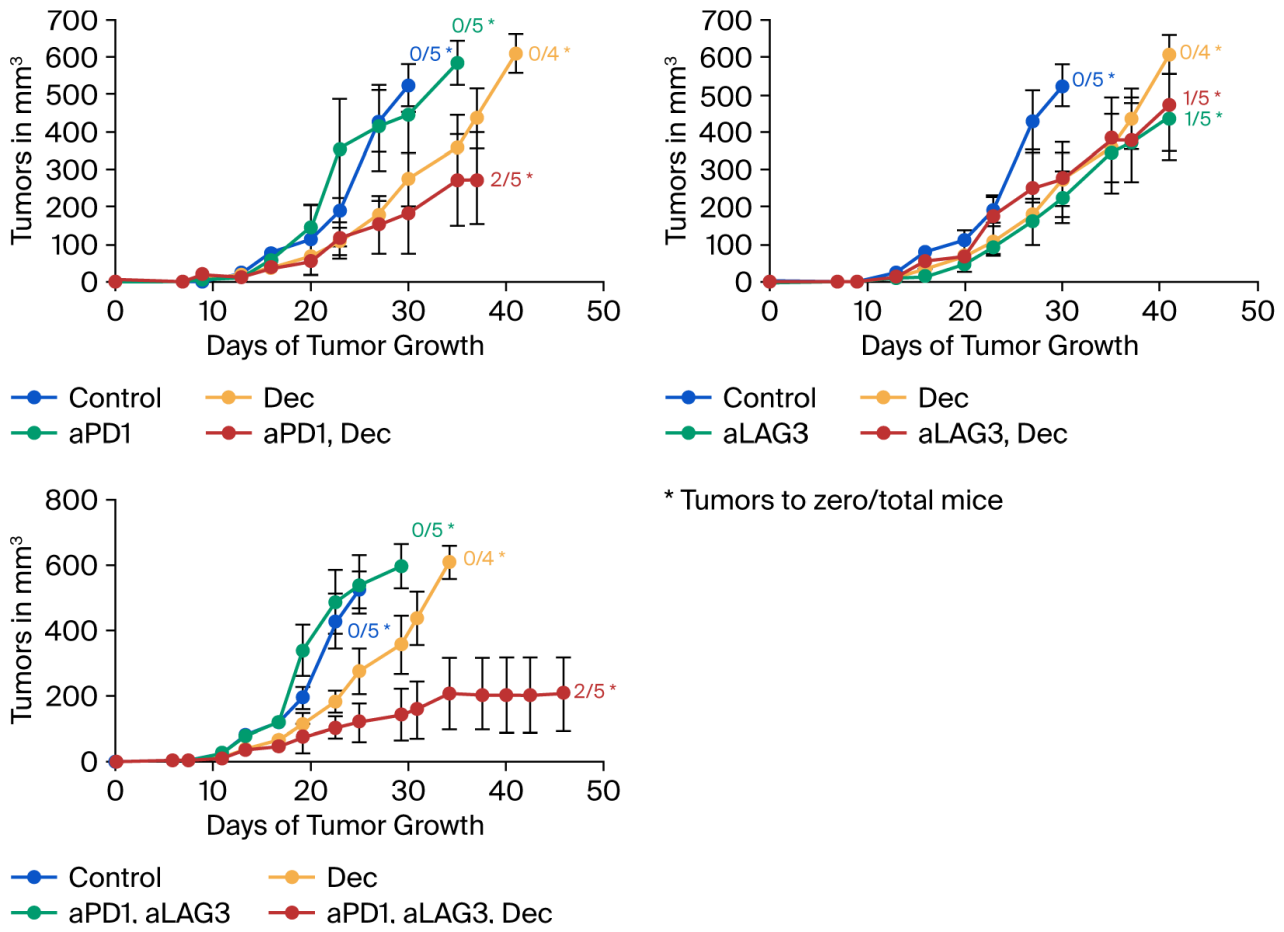
Balb/c mice inoculated with 4T1 tumor cells on day 0 were treated with Dec alone, aPD1 alone, aLAG3 alone, or combinations. Decitabine (1 mg/kg i.p.) was given on days 14–16, 21–23, and 27–29; anti-PD-1 and anti-LAG-3 (100ug) were given on days 16, 19, 22, 25, and 28. All three graphs were derived from the same experiment and share the same curves for control and Dec alone. Graphs were separated by treatment for legibility. Welch two-sample unpaired *t*-tests were used to compare the mean tumor volume and showed no significant difference in anti-PD-1 + anti-LAG3 +Dec compared to any group. The non-significant results should be interpreted cautiously given small group sizes and large uncertainty around the mean difference estimates.

**Figure 12:**
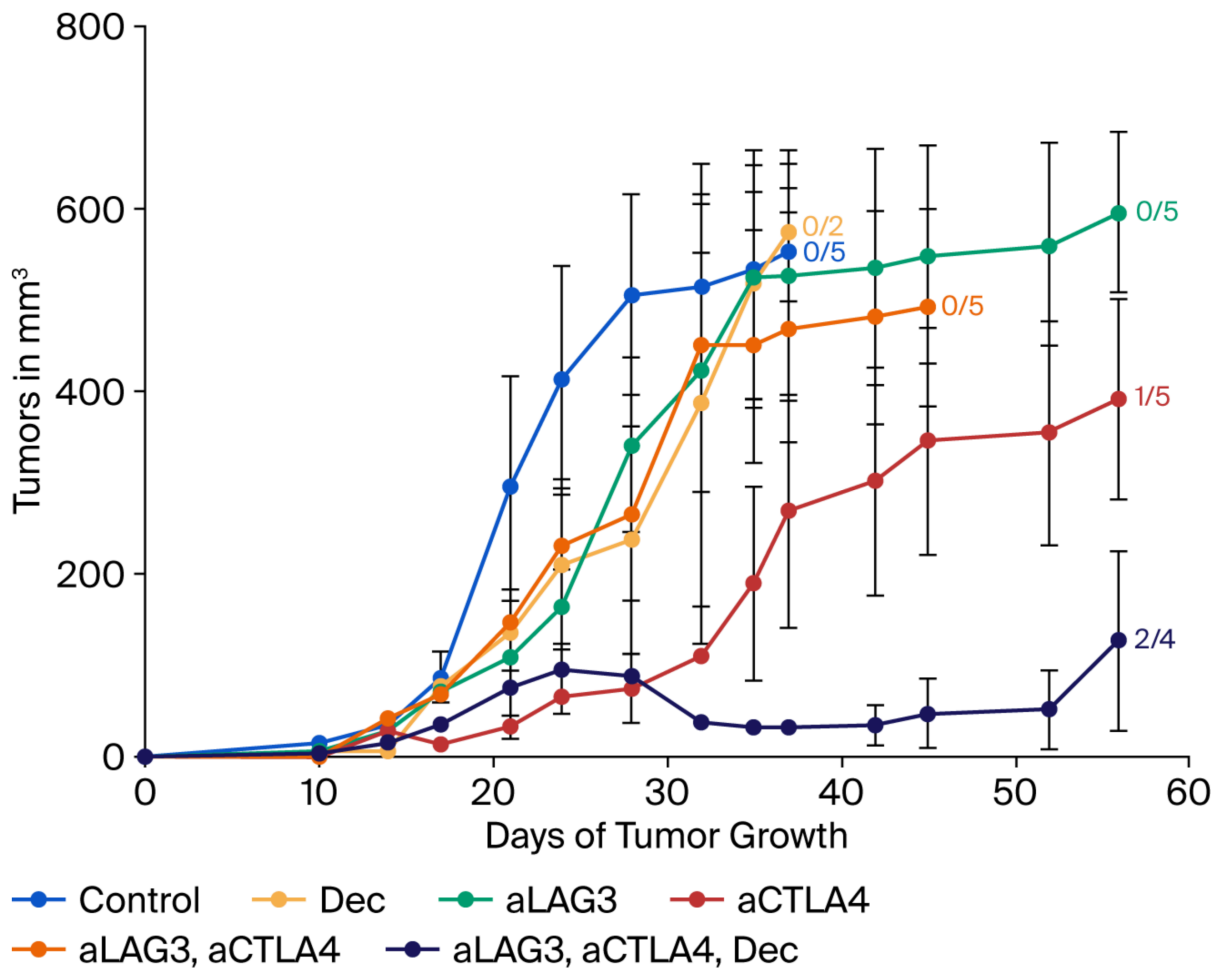
Balb/c mice inoculated with 4T1 tumor cells on day 0 were treated with Dec alone, aCTLA4 alone, aLAG3 alone, or combinations. Decitabine (1 mg/kg i.p.) was given on days 14–16, 21–23, and 27–29; anti-CTLA4 and anti-LAG-3 (100ug) were given on days 16, 19, 22, 25, and 28. Tumors in mice treated with aLAG3 + aCTLA4 + Dec showed significant tumor size reductions (*p* < 0.0001).

Given our findings of improved control of tumor growth using Dec + anti-LAG3-based dual ICB in E0771, we also investigated Dec + aLAG3-based dual-ICB therapy in 4T1 tumors. Unlike in our experiments with E0771, the treatment of 4T1 with Dec + aPD1 + aLAG3 led to a non-significant reduction in tumor burden compared to any group, as indicated by Welch two-sample unpaired *t*-tests. The non-significant results should be interpreted cautiously given the small group sizes and large uncertainty around the mean difference estimates (Figure 11). However, we did observe a greater reduction in tumor burden achieved with Dec + anti-CTLA4 + anti-LAG compared to controls or single-ICB-agent therapies with or without Dec (Figure 12).

## 4. Discussion

Despite the recent achievements in cancer immunotherapy, a subset of patients do not respond to ICB, and there are currently few reliable biomarkers for ascertaining who will or will not benefit from ICB, particularly in the setting of early breast cancer. Our group has previously shown that DNMTi treatment with Dec or Gua prevents the systemic accumulation of immunosuppressive cells and, when combined with adoptive cellular immunotherapy, significantly reduced tumor burden in the 4T1 and E0771 murine models of triple-negative breast cancer [19,21]. Interestingly, we have never observed any significant effect of DNMTi alone on tumor growth, in contrast to other reports [29]. It has been postulated that neoadjuvant immunotherapy could be advantageous compared to adjuvant immunotherapy by treating when tumor antigen and tumor-infiltrating T lymphocytes are still present in the TME and the T cells can respond to the rescue of immunosuppression by ICB [33]. Indeed, Liu et al. showed that neoadjuvant treatment with ICB prior to the resection of primary murine breast cancers was superior to the same treatment administered after tumor resection [34]. In this study, we have shown that combining Gua or Dec with a single low dose of cyclophosphamide and ICB is effective at inducing tumor regression in the 4T1 model and, when administered prior to the resection of primary tumors, improved metastasis-free survival. We found that this combination of treatments was more effective than any monotherapy or combination of dual therapies. This suggests that DNMTi acts synergistically with other immune therapies to enhance their anti-tumor efficacy. Several groups have proposed that low-dose cyclophosphamide (here, 100 mg/kg) acts as an immune modulator (e.g., by reducing Tregs, perhaps explaining its enhancement of metastasis-free survival [35,36]). We too have previously shown that low-dose cyclophosphamide can reduce the prevalence of Tregs in the tumor microenvironment, and that combined treatment with both Gua and low-dose cyclophosphamide significantly reduces the number of Tregs compared to control or either treatment alone [19]. Moreover, moderate depletion of the lymphoid compartment facilitates the expansion of adoptively transferred T cells [31,35]. Our findings that DNMTi, either Gua or Dec, can not only significantly augment tumor regression, but also improve metastasis-free survival, when combined with ICB, highlight that the mechanism by which DNMTi impacts the tumor microenvironment may be of interest for future research.

To determine whether the effect of DNMTi on immune therapy could be generalized to another TNBC model, we shifted our focus to the E0771 murine model. We found that adding DNMTi treatment restored the response to ICB for an ICB-resistant E0771 subline. Based on the findings of Luo et al. and our prior work with 4T1 [21], we initially hypothesized that the mechanism by which guadecitabine restores immune sensitivity to ICB in the E0771 model of TNBC could be the upregulation of MHC class I expression. Luo et al. found that treatment with guadecitabine demethylated the promoter for MHC class I, thus increasing its transcription and expression [37]. By serially selecting and passaging tumors that were resistant to dual-ICB treatment, we developed a stable subline of ICBR E0771 tumors. Initially, we expected that the ICBR E0771 tumor cells might have developed resistance to ICB by developing tumor-intrinsic MHC I downregulation. However, ICBR tumors exhibited a modest MHC I upregulation, not downregulation. We also expected that Gua would upregulate MHC class I expression and increase the activity of anti-tumor cytotoxic T cells, but we did not find significant changes there either.

The most reproducible finding associated with the DNMTi treatment of 4T1 and E0771 tumors was increased DC infiltration. DCs contribute to the anti-tumor response by priming naïve T cells and can present tumor antigens more efficiently than other myeloid-derived counterparts. We found that DNMTi-based therapies increased DC and T cell infiltration, namely naïve T cells of both CD8 and CD4 subsets. This further suggests a potential increase in T cell priming by DCs. DC availability is especially important in overcoming ICBR, because DC depletion is a key feature of “cold” tumors [38]. The specialized functions of DCs are guided by subtype; cDC1s are important for the response to ICB and activate both CD4 and CD8 T cells, while cDC2s mobilize CD8+ cells and promote NK cell expansion [39]. While we did not investigate the composition of DC subsets in our tumor analysis, this may be a future area of research interest.

While we did not further investigate the mechanism of DC accumulation, high numbers of tumor-infiltrating lymphocyte (TILs), as found in 4T1 models, are known to facilitate the entry of DCs into tumors [17,40]. Conversely, intratumoral DCs also initiate CD8+ TILs’ influx, and may explain the later increase in CD8+ cells that we found by flow cytometry [17]. Furthermore, MDSCs are also well-described to suppress the differentiation of myeloid progenitors into DCs [10,18]. Thus, it is possible that the reduction in MDSCs may have also reduced the block of myeloid progenitors’ differentiation into DCs, leading to the observed rise. The analysis of intratumoral natural killer (NK) cells from control mice vs. mice treated with triple therapy did not show significant differences on day 18 or 22.

Altogether, these findings point to a stepwise depletion of suppressive myeloid cells, DC accumulation, and T cell expansion and reversal of exhaustion. These changes may underlie the durable tumor regression along with metastasis-free survival benefit induced by triple therapy.

While both CD4+ and CD8+ T cells are capable of anti-tumor activity, CD8+ cells are traditionally considered the predominant subtype with direct cell-killing activity. Others have previously reported on the greater anti-tumor capability of central memory T cells as opposed to effector T cells [30,31]. In mice bearing 4T1 tumors treated with triple therapy, CD8+ T cells shifted toward the central memory subtype, suggesting that these cells may go on to prevent metastasis even after the treatment regimen has ended [35].

Some groups have suggested that neoadjuvant cytotoxic treatments prior to surgical resection may enhance anti-tumor immunity by releasing tumor antigens that stimulate T cell responses in both local and metastatic TMEs, leading to a more robust cell-mediated killing response [41,42]. Here, we show that neoadjuvant treatment with DNMTi and low-dose cyclophosphamide, plus neoadjuvant and adjuvant treatment with anti-PD-1 resulted in improved metastasis-free survival compared to any other monotherapy or dual-therapy group. This supports the findings of Liu et al., who found that treating TNBC with anti-PD-1 prior to surgical resection improved long-term survival in mice and stimulated the infiltration of effector T cells, which persisted long-term as memory T cells.

T cell exhaustion is a dysfunctional differentiation state that results from chronic antigen stimulation within the TME, and is characterized by reduced cytotoxic activity along with the upregulation of inhibitory receptors, including PD-1, CTLA-4, and LAG-3 [22,23,43]. The exhaustion of T cells is mediated by stable epigenetic events resulting in ICB resistance and a limited ability to regain effector function. Therefore, DNMTi inhibition may help reprogram exhausted T cells and restore anti-tumor immunity [22,23,44,45], as supported by our observations that IBCR in the E0771 murine model can be overcome with DNMTi-containing therapies. This allows for the development of a more robust Tcm population, thus conferring greater anti-tumor efficacy and increased likelihood of durable tumor control. Importantly, our finding that ICBR was overcome with DNMTi-containing therapies was reproducible with two different combinations of dual-ICB (aPD1 + aCTLA4 and aPD1 + aLAG3), but not with single-agent ICBs alone. This suggests that 4T1 and E0771 tumors may employ redundant checkpoint pathways, and thus, the effective functional rescue of exhausted T cells requires simultaneously blocking multiple checkpoint pathways.

There has also been interest in enhancing anti-tumor efficacy through anti-LAG3-based dual ICBs, namely aLAG3 combined with aPD1 based on the co-expression of LAG3 and PD1 in the TME [46,47,48,49]. Like PD1, LAG3 is a checkpoint inhibitor that inhibits T cell activation and is upregulated during Treg exhaustion [49]. As demonstrated in the phase 3 RELATIVITY-047 trial, combination therapy of aLAG3 + aPD1 significantly extended progression-free survival in patients with metastatic melanoma when compared to aPD1 therapy alone [50]. As such, we also tested aLAG3 dual ICB combined with DNMTi in the setting of TNBC. We showed that adding DNMTi drugs to dual ICB dramatically improved the anti-tumor effects in both E0771 and 4T1 TNBC models.

There are several limitations to this study. Although DNMTi-containing therapy restored the responsiveness to ICB and was associated with increased DC and T cell infiltration, the exact mechanisms through which DNMTi modifies immune compartments of the TME are unclear. Our analysis also did not include the distinction of DC subsets or specific identification of iNKT cells, which may be important contributors to the observed reduction in tumor burden. Similarly, functional assays were not performed to confirm the reversal of T cell exhaustion or expansion of central memory T cells.

Altogether, our findings suggest that DNMT inhibition can enhance ICB efficacy and restore responsiveness to ICB in murine models of triple-negative breast cancer that are resistant to ICB alone, even dual ICB. DNMTi-containing ICB regimens improved tumor regression and metastasis-free survival and were associated with increased DC and T cell infiltration along with the expansion of Tcms, suggesting enhanced immune priming and durable anti-tumor immunity. These findings support the further investigation of combination therapy with DNMTi and dual ICB as a strategy to overcome ICB resistance in TNBC. Indeed, we have completed and reported on a neoadjuvant trial in which women with locally advanced breast cancer received Dec followed by two doses of pembrolizumab prior to starting neoadjuvant chemotherapy. This resulted in an increase in stromal TIL and PD-L1 expression [51].

## 5. Conclusions

This study demonstrates that DNMT inhibition can enhance ICB efficacy and restore responsiveness to ICB in two murine models of TNBC which are resistant to single or dual agent ICB. DNMTi agents decitabine and guadecitabine both produced durable anti-tumor responses in both the 4T1 and E0771 models. While mechanisms of tumor suppression are unclear, tumors of mice treated with DNMTi-containing therapy showed reduction in immunosuppressive myeloid populations, increased dendritic cell infiltration, expansion of central memory CD8^+^ T cells, and reduction in T cell exhaustion. These findings support the use of DNMTis as a method of re-sensitizing ICBR tumors and provide a strong rationale for the incorporation of DNMTis into ICBR clinical treatment regimens. Early clinical studies of adding Dec and pembrolizumab treatment prior to neoadjuvant chemotherapy in women with locally advanced BC showed increased stromal TILs and PD-L1 expression, which further supports the translational potential of this approach. Further clinical investigations are needed to understand optimal dosing and patient populations who may benefit from treatment with DNMTi-containing ICB.

## Data Availability

The original contributions presented in this study are included in the article/Supplementary Material. Further inquiries can be directed to the corresponding author.

## Supplementary Materials

The following supporting information can be downloaded at: https://www.mdpi.com/article/10.3390/cancers18152480/s1, Figure S1: Viable, single cells were gated on CD45+ cells and then gated for additional immune cell populations; Figure S2: Viable, single cells were gated on CD45- to exclude immune cells and then analyzed for H2Kb and H2Db expression.

## Abbreviations

The following abbreviations are used in this manuscript:

ICB	Immune-checkpoint blockade
Gua	Guadecitabine
Dec	Decitabine
Cyp	Cyclophosphamide
TME	Tumor microenvironment
DNMTi	DNA methyltransferase inhibitor
TNBC	Triple-negative breast cancer
Tcm	Central memory T cells
DC	Dendritic cells
ER	Estrogen receptor
PR	Progesterone receptor
HER2	Human epidermal growth factor receptor
CTL	Cytotoxic T lymphocytes
MDSC	Myeloid-derived suppressor cells
cDC	Conventional dendritic cells
Treg	Regulatory T cells
mfp	Mammary fat pad
TIL	Tumor-infiltrating lymphocyte
Teff	Effector T cells
ICBR	Immune-checkpoint blockade-resistant
IP	Intraperitoneally
MFI	Mean Fluorescence Intensity
iNKTs	Invariant natural killer cells
PD-1	Programmed cell death protein 1
CTLA-4	Cytotoxic T-Lymphocyte-Associated Protein 4
LAG3	Lymphocyte-activation gene 3
