# Supplementary material for: DNA Methyltransferase Inhibitors, Decitabine and Guadecitabine Overcome Immune-Checkpoint Blockade Resistance and Achieve Tumor Regression in the E0771 and 4T1 Murine Models of Triple-Negative Breast Cancer
Source: Cancers (Basel). 2026 Aug 2;18(15):2480. doi: 10.3390/cancers18152480 (PMC13464779; doi:10.3390/cancers18152480)
Supplement: Supplementary file 1 [file cancers-18-02480-s001.zip › cancers-4422898-supplementary.pdf]

Supplemental Figures

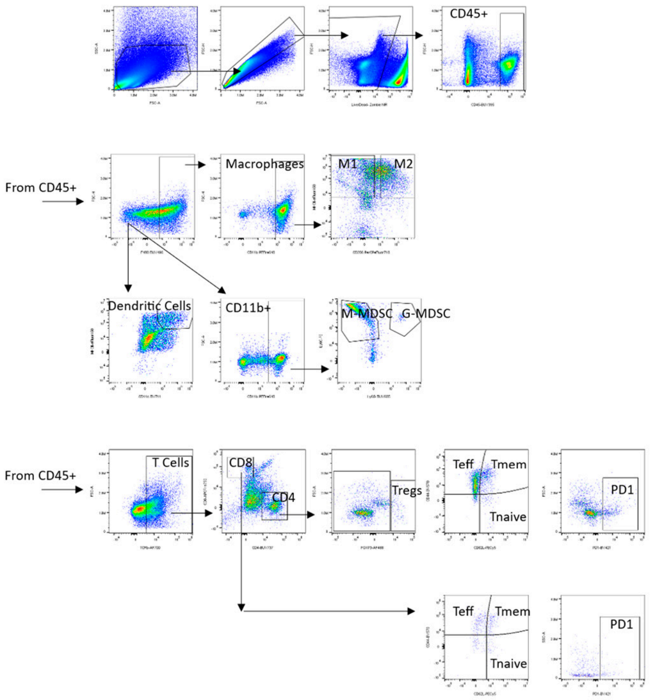

Figure S1. Viable, single cells were gated on CD45+ cells and then gated for additional immune cell populations.

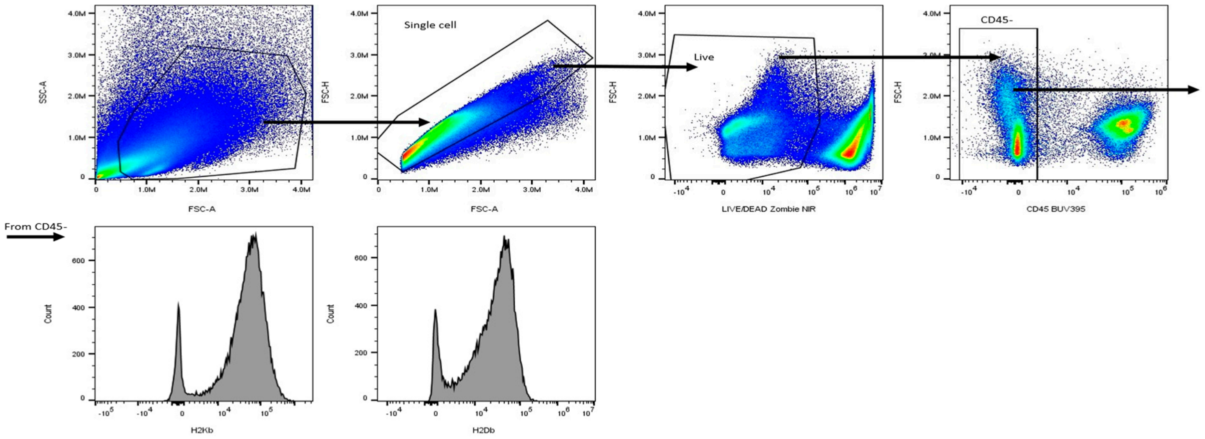

Figure S2. Viable, single cells were gated on CD45- to exclude immune cells and then analyzed for H2Kb and H2Db expression.

For additional information of gating methods, see: Isbell MG, Wendling A, Pei X, Kumar A, Mannangatti P, Krisanits BA, Cheatham S, Michenkova M, Shumilov K, Mendoza RG, et al. Making deep immunophenotyping accessible: the successful application of a guided 23-parameter mouse immunophenotyping panel package provided through a shared resource. Front Immunol. 2026 Jan 9;16:1668405. doi: 10.3389/fimmu.2025.1668405. PMID: 41583432; PMCID: PMC12827670.
